# Entropy and topological indices of nanostar dendrimers: a graph-theoretic approach

**DOI:** 10.3389/fchem.2026.1858907

**Published:** 2026-07-29

**Authors:** Allah Nawaz, Syed Ahtsham Ul Haq Bokhary, Muhammad Imran

**Affiliations:** 1 Centre for Advanced Studies in Pure and Applied Mathematics, Bahauddin Zakariya University, Multan, Pakistan; 2 Department of Electrical Engineering, Prince Mohammad Bin Fahd University, Al Khobar, Saudi Arabia

**Keywords:** degree distance index, entropy, Gutman index, nanostar dendrimers, Wiener index

## Abstract

Nanostar dendrimers have received considerable attention due to their unique properties and various applications, such as drug delivery, nanomedicine, and materials science. A comprehensive analysis of their structural characteristics is essential for optimizing these capabilities. In this study, we consider infinite families of nanostar dendrimers to analyze and characterize their structural properties. Graph theory provides a mathematical framework to examine the connectivity and topological features of these complex nanostructures. Particularly, we compute several key topological indices, including the degree distance, Gutman, Wiener, and Wiener polarity indices, for this class of nanostar dendrimers. Furthermore, we present a graphical analysis to visually determine the results, providing a clearer understanding of the structural trends and patterns within the nanostar dendrimers. Additionally, we extend our analysis by determining the graph entropy, which serves as a measure of the structural complexity and the information embedded within the dendrimer structure.

## 1 Introduction

Chemical graph theory is a branch of mathematical chemistry that plays an important role in the development of the chemical sciences. Molecular graphs can be represented in various forms, including polynomial expressions, matrices, or a single numeric value that encapsulates the entire graph. These representations are essential for correlating the atomic arrangement of a compound with its physicochemical properties or biological activity (Gutman, 2013; Gross et al., 1998). Topological indices are numerical invariants derived from molecular structures that encode its structural features with broad applications in theoretical chemistry. These indices serve as powerful tools for predicting the diverse physicochemical properties of chemical compounds with varying molecular architectures. Among the most significant types are distance-based, degree-based, and spectrum-based indices. Degree-based topological indices, in particular, have gained substantial attention in mathematical chemistry. They are defined based on the degrees of the vertices within a graph, as discussed in more detail in Arockiaraj and Clement (2020), Ashraf et al. (2022), and Dobrynin and Kochetova (1994). Researchers used different approaches to find these indices, but in our study, we focus on distance-based topological indices; refer Malik et al. (2023) for more details.

Carbon nanostructures have found many potential industrial applications, such as energy storage, gas sensors, biosensors, nanoelectronic devices, and chemical probes (Farooq and Malik, 2015). Nanostar dendrimers have emerged as promising nanomaterials due to their well-defined branched architectures, tunable sizes, and versatile surface functionalities. These qualities make them attractive for a number of uses, such as gene therapy, targeted drug administration, and imaging (Zeng and Zimmerman, 1997). Structurally, nanostar dendrimers are hybrid nanoparticles formed by combining gold nanostars and dendrimers. Gold nanostars are selected for their high surface area and unique optical properties, which make them effective for sensing and imaging applications. In contrast, dendrimers are chosen due to their ability to carry multiple drug molecules and target specific cells or tissues in the body.

The researchers initially synthesized the gold nanostars using a seed-mediated growth method to create the nanostar dendrimers. They then functionalized the surface of the gold nanostars with a dendritic molecule known as the polyamidoamine (PAMAM) dendrimer, which has numerous functional groups on its surface that are suitable for attaching drug molecules (Tomalia and Frechet, 2002). The produced nanostar dendrimers exhibit a core-shell structure, with the gold nanostars forming the core and the dendrimers forming the shell. Previous studies have demonstrated that these hybrid nanoparticles successfully delivered drug molecules to cancer cells *in vitro* and could also be utilized for imaging applications (Hosoya, 1971; Wiener, 1947). This study not only contributes to the field of molecular graph theory, enabling a deeper understanding and utilization of the unique properties of nanostar dendrimers, but also contributes to understanding the underlying topology of the graph.

Nanostar dendrimers exist in several structural forms. The first two were introduced by Dorosti et al. (2009), where the Cluj index was calculated, a topological metric that quantifies the proximity of every vertex within a molecular graph. Bokhary et al. (2024) computed some indices using a vertex-weighted graph for the first type. The third type of nanostar dendrimers was introduced by Farahani et al. (2017). For this graph, some topological indices, such as the Zagreb indices and the topological connectivity indices, have been computed by Farahani (2015). The Hosoya index of some nanostar dendrimers was computed by Movahedi et al. (2024). Many topological indices and entropy for some families of nanostar dendrimers were also calculated by Qummer et al. (2026), Sattar et al. (2021), and Siddiqui et al. (2018). The energy of some tree dendrimers was calculated by Bokhary and Tabassum (2022), while additional results on the topological indices of nanostar dendrimers can be found in Gao et al. (2018), Hui et al. (2022), Iqbal et al. (2020), and Iqbal et al. (2019).

The present study investigates the third type of nanostar dendrimer $D_{3}\left[n\right]$ . The new results are the recursive formulae for $W\left(D_{3}\left[n\right]\right)$ , $Gut\left(D_{3}\left[n\right]\right)$ , $DD\left(D_{3}\left[n\right]\right)$ , $W_{P}\left(D_{3}\left[n\right]\right)$ , and the degree-distribution entropy $Ent\left(D_{3}\left[n\right]\right)$ . Earlier work on related families includes the Wiener and Wiener polarity indices for the first type in Bokhary et al. (2024) and Zagreb and connectivity indices for the third type in Farahani et al. (2017) and Farahani (2015). These studies do not provide the closed recursive formulae for the Gutman index, degree distance index, and degree-distribution entropy obtained here for $D_{3}\left[n\right]$ .

The structure of the paper is as follows: Section 1 introduces the general background and outlines the purpose of the study. Section 2 reviews the fundamental ideas of graph theory, dendrimers, and topological indices. The primary results and their discussion are presented in Section 3. In Section 4, the entropy of the graph is computed. Section 5 provides a graphical analysis to visually determine the results and highlight structural trends and patterns. Finally, Section 6 presents the concluding remarks.

## 2 Basic concepts and literature review

Let G=(V(G),E(G)) be a simple connected graph, with a vertex set $V=V\left(G\right)=\left\{v_{1},v_{2},\ldots ,v_{n}\right\}$ and an edge set $E\left(G\right)=\left\{e_{1},e_{2},\ldots ,e_{n}\right\}$ . For a vertex $v_{i}\in V\left(G\right)$ , the degree of a vertex $v_{i}$ , denoted by $\text{deg}\left(v_{i}\right)$ , corresponds to the number of edges incident with $v_{i}$ . The maximum and minimum degrees of a graph G are denoted by Δ and δ , respectively. For a graph G , the distance between two vertices $v_{i}$ and $v_{j}$ , written as $d\left(v_{i},v_{j}\right)$ , is defined as the length of the shortest path between them.

### 2.1 Topological indices

Topological indices are numerical values derived from the molecular structure of chemical compounds. They provide a simplified representation of a molecule’s topology or topological connectivity without considering its spatial arrangement. These indices are useful tools in computational chemistry, drug design, and materials science due to their ability to provide valuable insights into the structure–property relationships of chemical compounds (Arockiaraj and Clement, 2020; Bokhary et al., 2022). They allow researchers to extract meaningful information from the molecular structure, enabling the efficient screening, prediction, and design of molecules with desirable properties for various application; refer Duncan and Izzo (2005) and Gutman (2013) for more details. In this article, we use the following standardized notation: W(G) for the Wiener index, $W_{P}\left(G\right)$ for the Wiener polarity index, Gut(G) for the Gutman index, DD(G) for the degree distance index, and Ent(G) for the graph entropy.

### 2.2 Wiener and Wiener polarity indices

The Wiener index, denoted as W(G) , was first introduced by Wiener (1947) and is defined as the sum of all pair-wise shortest path distances between distinct vertices. It is a valuable topological descriptor widely used in quantitative structure–activity relationship (QSAR) studies and chemical database mining (Gutman and Körtvélyesi, 1995). Additionally, it has applications in the field of chemistry, particularly in the study of molecular graphs. There is a rich history and work on the Wiener index of various graphs (Dobrynin et al., 2001; Klavžar and Nadjafi-Arani, 2014). Mathematically,

$$W\left(G\right)=\frac{1}{2}\underset{u,v\in V\left(G\right)}{\sum }d\left(u,v\right),$$

where d(u,v) represents the shortest path length between vertices u and v in the graph G .

The Wiener polarity index is a modification of the Wiener index that considers the type of chemical bonds in addition to their lengths. It is denoted by $W_{P}\left(G\right)$ and is defined as the cardinality of the set of vertex pairs at distance three in G . Mathematically,

$$W_{P}\left(G\right)=\left\{\left(u,v\right)|d\left(u,v\right)=3,\;u,v\in V\left(G\right)\right\}.$$

Hosoya (1971) found a physical–chemical interpretation of $W_{P}\left(G\right)$ . Actually, the Wiener polarity index of different kinds of graphs has been studied, such as trees, unicyclic and bicyclic, hexagonal systems, and lattice network graphs; refer Klavžar et al. (1995) and Kowalski et al. (2019) for more details.

### 2.3 Gutman and degree distance indices

Das et al. (2015) and Gutman (2013) introduced the index now known as the Gutman index $Gut\left(G\right)$ , for a connected graph G . It is defined as follows:

$$Gut\left(G\right)=\frac{1}{2}\underset{u,v\in V\left(G\right)}{\sum }\left(deg\left(u\right).deg\left(v\right)\right)d\left(u,v\right),$$

where $deg\left(u\right),deg\left(v\right)$ is the degree of the vertices u and v , respectively, and d(u,v) represents the distance between vertices u and v in the graph G . Recently, many authors have investigated this index for a wide range of graph families, including trees, dendrimers, and fractal.

The degree distance index was discussed by Dobrynin and Kochetova (1994). The degree distance index denoted by $DD\left(G\right)$ of a connected graph G is the product of the sum of the degree of the vertices and the distance between these vertices. Mathematically, it is defined as

$$DD\left(G\right)=\frac{1}{2}\underset{u,v\in V\left(G\right)}{\sum }\left(deg\left(u\right)+deg\left(v\right)\right)d\left(u,v\right),$$

where $deg\left(u\right),deg\left(v\right)$ is the degree of the vertices u and v , respectively, and d(u,v) is the distance between vertices u and v in the graph G .

## 3 Results and discussions

**FIGURE 1:**
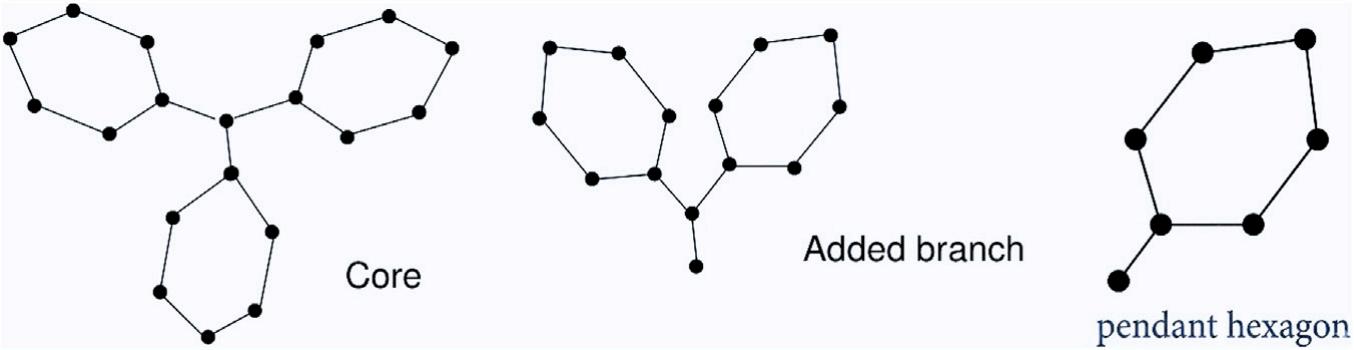
Core, added branch, and the pendant hexagon of the dendrimer.

In this study, we use topological indices and graph theory to understand the structural characteristics of nanostar dendrimers. We represent nanostar dendrimers as graphs, with the central core, shown in Figure 1, as the central vertex and dendritic branches as peripheral vertices. The edges connecting these vertices signify the covalent bonds between different segments of the dendrimer.

### 3.1 The third type of nanostar dendrimers

**FIGURE 2:**
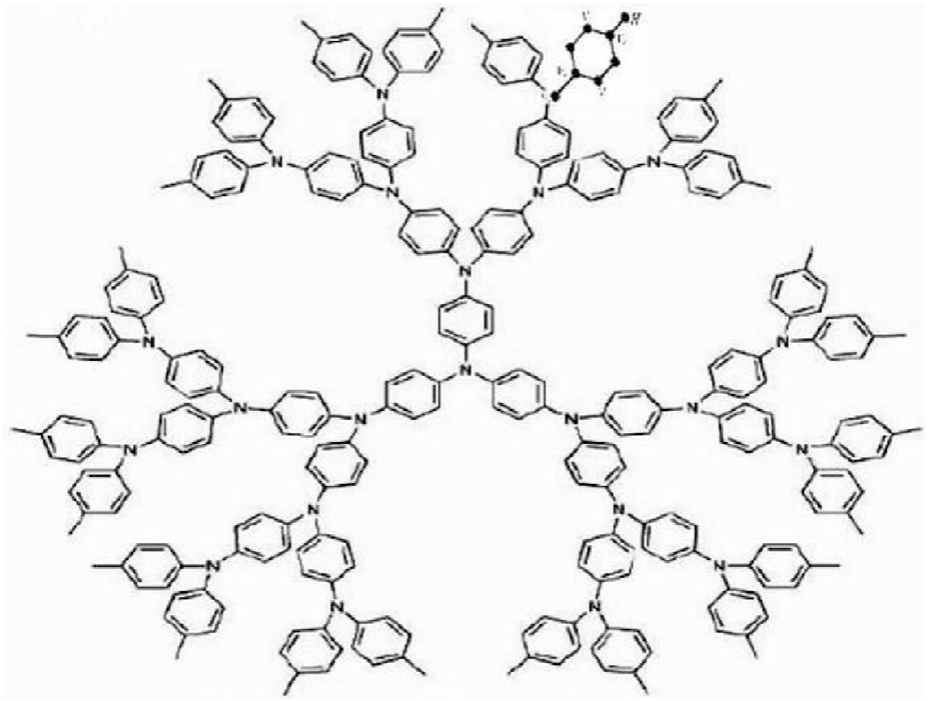
Third type of nanostar dendrimers growing up to three stages.

We first introduce the construction of the nanostar dendrimers. Let $D_{3}\left[n\right]$ denote the graph obtained after n growth stages. For n=0 , three hexagons share a common vertex named as core, as shown in Figure 1. Let the added branch (H) denote the hexagonal branch in Figure 1, with |V(H)|=14 and |E(H)|=15 . For n≥1 , the graph $D_{3}\left[n\right]$ is obtained from $D_{3}\left[n-1\right]$ by attaching $3\cdot 2^{n-1}$ disjoint copies of H to the pendant vertices of stage n−1 . The hexagons added at stage i are known as pendant hexagons. Their numbers at stages 0,1,2,…,n are $3,6,12,\ldots ,3\cdot 2^{n}$ , and the total number of hexagons in $D_{3}\left[n\right]$ is $3\left(2^{n+1}-1\right)$ . For n=3 , Figure 2 illustrates $D_{3}\left[3\right]$ after three growth stages; the core in Figure 1 corresponds to stage 0 in Figure 2.

For non-negative integers n , a direct count gives $|V\left(D_{3}\left[n\right]\right)|=39\cdot 2^{n}-20$ and $|E\left(D_{3}\left[n\right]\right)|=45\cdot 2^{n}-24$ . For n≥0 , every vertex has degrees 2 or 3. Let $V_{1}=\left\{x\in D_{3}\left[n\right]:\deg\left(x\right)=2\right\}$ , $V_{2}=\left\{x\in D_{3}\left[n\right]:\deg\left(x\right)=3\right\}$ , and $V\left(D_{3}\left[n\right]\right)=V_{1}\cup V_{2}$ . The degree partition is

$$|V_{1}|=27\cdot 2^{n}-12,\;|V_{2}|=12\cdot 2^{n}-8.$$

Indeed, $2|V_{1}|+3|V_{2}|=2|E\left(D_{3}\left[n\right]\right)|$ for all n≥0 . Let $\rho_{n}=3\cdot 2^{n}$ denote the number of pendant hexagons at stage n . The sets $V_{1}\left(\rho \right)$ and $V_{2}\left(\rho \right)$ denote the vertices of degrees 2 and 3 on these pendant hexagons, respectively. The maximum distance between pendant hexagons at stage i is 10i+2 , where i≥0 .

Some details about Figure 2 that are helpful in the computations are as follows:

1. For k≥0 , let $I_{n,\;5k-3}$ denote the number of hexagons whose graph distance from a fixed pendant hexagon of stage n equals 5k−3 .
2. For a newly attached pendant hexagon at stage n , let $a_{k}$ , $b_{k}$ , and $c_{k}$ denote the total contributions to the indices, arising from pairs of vertices separated by distance 5k−3 and involving vertices of the types $\left(V_{1},V_{1}\right)$ , $\left(V_{2},V_{2}\right)$ , and $\left(V_{1},V_{2}\right)$ , respectively.
3. Let $a_{k}$ and $b_{k}$ denote the distance from the vertices of degrees 2 and 3, respectively, of the pendant hexagons to the vertices of degrees 2 and 3 of hexagons located at a distance of 5k−3 . Here, $a_{0}$ denotes the distance between the vertices of degree 2 of the pendant hexagon, as shown in Figure 1. The base distances are $a_{0}=12+6,a_{1}=2\left(4\times 3+6\right)+2\left(4\times 4+6\right)+4\times 5+6,a_{2}=2\left(4\times 8+6\right)+2\left(4\times 9+6\right)+4\times 10+6,a_{3}=2\left(4\times 13+6\right)+2\left(4\times 14+6\right)+4\times 15+6$ , and simplifying the pattern yields $a_{k}=2\left(4\times \left(5k-2\right)+6\right)+2\left(4\times \left(5k-1\right)+6\right)+4\times 5k+6=100k+6$ . Similarly, $b_{0}$ is the distance between the vertices of degree 3 of the pendant hexagon illustrated in Figure 1. $b_{0}=0,b_{1}=1+2,b_{2}=33=30\times \left(k-1\right)+3,b_{3}=63=30\times \left(k-1\right)+3,\ldots ,b_{k}=30\times \left(k-1\right)+3$ .
4. Let $c_{k}$ represent the distance from the degree-2 vertices of the pendant hexagons to the degree-3 vertices of the hexagons at a distance of 5k−3 and the vertex of degree 3 joining two hexagons to the degree-2 vertices of hexagons at a distance of 5k−3 . $c_{0}=3+2\left(2+1\right)=9,c_{1}=4+2\left(3+2\right)+5+2\left(4+3\right)=33=50k-17,c_{2}=9+2\left(8+7\right)+10+2\left(9+8\right)=83=50k-17,c_{3}=14+2\left(13+12\right)+15+2\left(14+13\right)=133,\ldots ,c_{k}=50k-17$ . Thus, for k≥1 , the shortest-path distances appearing in the contribution counts of the added branch are given by $a_{k}=100k+6,b_{k}=30k-27,c_{k}=50k-17$ .

Table 1 lists the first values of $a_{k}$ , $b_{k}$ , and $c_{k}$ . For the following summations, Lemma 3.1.2 applies to odd k with 1≤k≤2n−1 , Lemma 3.1.3 applies to even k with 2≤k≤2n , and Lemma 3.1.4 gives the maximum-distance case at k=2n+1 .

**TABLE 1:** Distance contributions used in the recursive index formulae.

k	$a_{k}$	$b_{k}$	$c_{k}$
0	18	0	9
1	106	3	33
2	206	33	83
3	306	63	133

Lemma 3.1.1 *For* 1≤i≤n *,*

$$\rho_{i}=2.\rho_{i-1}=3.2^{i}.$$

Proof. The graph’s construction shows that the number of pendent hexagons at the $i^{th}$ stage is twice the number of pendent hexagons at the ${\left(i-1\right)}^{th}$ stage. Therefore, we obtain

$$\rho_{i}=2.\rho_{i-1}.$$ (3.1.1)

by backward substitution, and using $\rho_{0}=3$ , Equation 3.1.1 reduces to $\rho_{i}=2^{i}.\rho_{0}=3.2^{i}$ .

Lemma 3.1.2 *For the positive integer* n *, let* $I_{n,5k-3}$ *be the number of hexagons at a distance of* 5k−3 *, where* k *is odd from the pendant hexagons* $\rho_{n}$ *. Then,*

$$I_{n,5k-3}=9.2^{n+\frac{k-3}{2}}.$$

Proof. In Figure 2, if k=1 there are nine hexagons in the first stage at distance 2 from the pendant hexagons, so $I_{1,2}=9$ , and there are 18=9.2 hexagons in the $2^{nd}$ stage at distance 2 from the pendant hexagons, so $I_{2,2}=9.2$ and 36 for k=3 , $I_{2,12}=9.2^{2}$ . For n=3 , there are 36, 72, and 144 hexagons for k=1,3,5 accordingly, i.e., $I_{3,2}=9.2^{2}$ , $I_{3,12}=9.2^{3}$ , and $I_{3,22}=9.2^{4}$ . Similarly, for n=4 , the number of hexagons for each k=1,3,5,7 are $I_{4,2}=9.2^{3}$ , $I_{4,12}=9.2^{4}$ , $I_{4,22}=9.2^{5}$ , and $9.2^{6}$ at distance 32. Consequently, substituting odd values of k , the number of hexagons at distance 5k−3 from the pendent hexagons is $9.2^{n}.2^{\frac{k-3}{2}}$ , and we can write it as

$$I_{n,5k-3}=9.2^{n+\frac{k-3}{2}}.$$

Lemma 3.1.3 *For even positive integer* k *, let* $I_{n,5k-3}$ *be the number of hexagons at distance* 5k−3 *, where* 1≤k≤2n *, where* n=1,2,3,… *, from the pendant hexagons* $\rho_{n}$ *. Then,*

$$I_{n,5k-3}=3.2^{n+\frac{k}{2}}.$$

Proof. The construction of the graph implies that the number of hexagons at n stage that are at distance 5k−3 , where 2≤k≤2n and n≥1 , can be obtained from the pendent hexagons at the n−1,5k−3 stage and from the previous stage. Each pendent hexagon at n stage induces twice the number of hexagons as the hexagons of the previous stage, denoted as $2\left(3.2^{n+\frac{k}{2}-1}\right)$ hexagons. This implies that $I_{n,5k-3}=2\left(3.2^{n+\frac{k}{2}-1}\right)$ . By substituting $I_{1,7}=3.2^{2}$ and $I_{2,17}=3.2^{2+2},i.e.3.2^{n+k/2}$ . This implies that

$$I_{n,5k-3}=3.2^{n+\frac{k}{2}}.$$

Lemma 3.1.4 *For the positive integer* n *, let* $I_{n,10n+2}$ *be the number of hexagons at distance* 10n+2 *. Then,*

$$I_{n,10n+2}=3.2^{n}.$$

Proof. Figure 2 shows that there are 6 and 12 hexagons at the maximum possible distance (12 & 22) of stages 1 and 2, respectively. Furthermore, for n=3 , 24 hexagons are present at distance 32, which are all obtained from the ${\left(n-1\right)}^{th}$ stage with multiple 2. Therefore, for i≥2 , we obtain

$$I_{n,10n+2}=2.I_{n-1,10n-8}.$$ (3.1.2)

by backward substitution, and using $n_{1,12}=3.2$ , Equation 3.1.2 reduces to $I_{n,10n+2}=3.2^{n}$ .

### 3.2 Computation of the Wiener index of the graph $D_{3}\left[n\right]$

In the following theorem, the Wiener index of the graph $D_{3}\left[n\right]$ is computed.

Theorem 3.2.1 *Let* n *be a positive integer; then,*

$$W\left(D_{3}\left[n\right]\right)=4860.n4^{n}-4563.4^{n}+5070.2^{n}+W\left(D_{3}\left[n-1\right]\right).$$

Proof. Let $V_{1}$ and $V_{2}$ be the vertices of $D_{3}\left[n\right]$ of degrees 2 and 3 as defined above, respectively. Let $W\left(D_{3}\left[n\right]\right)=W_{1}\left(D_{3}\left[n\right]\right)+W_{2}\left(D_{3}\left[n\right]\right)+W_{3}\left(D_{3}\left[n\right]\right)$ , where $W_{1}$ , $W_{2}$ , and $W_{3}$ denote the contributions from pairs in $V_{1}\left(\rho_{n}\right)\times V_{1},V_{2}\left(\rho_{n}\right)\times V_{2},V_{1}\left(\rho_{n}\right)\times V_{2}$ , respectively, together with the corresponding contributions from $D_{3}\left[n-1\right]$ . Furthermore, suppose that $\rho_{i}$ are the pendent cycles at the $i^{th}$ stage with vertex sets $V_{1}\left(\rho_{i}\right)$ and $V_{2}\left(\rho_{i}\right)$ of degree 2 & 3 , respectively. For k≥2 , $I_{n,5k-3}$ represents the number of hexagons at distance 5k−3 in the $k^{th}$ stage, where 1≤k≤2n+1 from the pendant hexagons.

Let $a_{k}$ and $b_{k}$ denote the distance between the vertices of degree 2 and 3, respectively, of pendant hexagons to the vertices of degrees 2 and 3 of hexagons that are at a distance of 5k−3 . Additionally, $c_{k}$ represents the distance between the degree-2 vertices of pendant hexagons and the degree-3 vertices of hexagons at a distance of 5k−3 and the vertex of degree 3 joining two hexagons to the degree 2 vertices of hexagons at a distance of 5k−3 . Therefore, the total number of hexagons at the $n^{th}$ stage is $3.2^{n}$ , denoted as $I_{n,1}=3.2^{n}$ . In addition, $\forall n\geq 1,I_{n,7}=3.2^{n+1}$ , $\forall n\geq 2,I_{n,17}=3.2^{n+2}$ , $\forall n\geq 3,I_{n,27}=3.2^{n+3}$ , $\forall n\geq 4,I_{n,37}=3.2^{n+4}$ , and so on. Now, for n≥1 , 2≤k≤2n , by substituting these values recursively, we obtain $I_{n,5k-3}=3.2^{n+\frac{k}{2}}$ . Furthermore, $\forall n\geq 1,I_{n,2}=9.2^{n-1}$ , $\forall n\geq 2,I_{n,12}=9.2^{n}$ , $\forall n\geq 3,I_{n,22}=9.2^{n+1}$ , $\forall n\geq 4,I_{n,32}=9.2^{n+2}$ , and so on. Therefore, for n≥1 , 1≤k≤2n−1 , by substituting these values recursively, we obtain $I_{n,5k-3}=9.2^{n+\frac{k-3}{2}}$ . Furthermore, $I_{1,12}=12=3.4,I_{2,22}=48=3.4^{2},I_{3,32}=192=3.4^{3}$ and $I_{4,42}=768=3.4^{4}$ and so forth. Thus, when n≥1 , k=2n+1 , through recursive substitution, we obtain $I_{n,5k-3}=3.4^{n}$ .

Now, for $x\in V_{1}\left(P\right),y\in V_{1}$ , by counting the shortest-path distances between the corresponding vertex pairs, the Wiener contribution becomes $W_{1}\left(D_{3}\left[n\right]\right)=a_{0}.I_{n,1}+\left(\sum_{k=1}^{2n+1}\left(a_{k}\right)I_{n,5k-3}\right)=a_{0}.I_{n,1}+\left(\sum_{k=1}^{2n+1}\left(a_{k}\right)I_{n,5k-3}+\sum_{k=2}^{2n}\left(a_{k}\right)I_{n,5k-3}\right)+W_{1}\left(D_{3}\left[n-1\right]\right)$ . From Figure 1, we can compute that $a_{0}=18$ , and substituting $a_{k}=100k+6$ and using Lemmas 3.1.1, Lemmas 3.1.2, … , Lemmas 3.1.4, we obtain $W_{1}\left(D_{3}\left[n\right]\right)=18.3.2^{n}+\sum_{k=1}^{2n-1}9\left(100k+6\right)\left(2^{n+\frac{k-3}{2}}\right)+\sum_{k=2}^{2n}3\left(100k+6\right)\left(2^{n+\frac{k}{2}}\right)+\left(200n+106\right).3.4^{n}+W_{1}\left(D_{3}\left[n-1\right]\right).$ For ease, we can write it as

$$\begin{array}{ll} W_{1}\left(D_{3}\left[n\right]\right)=54.2^{n}+3.2^{n}\left(\sum_{k=1}^{2n-1}3\left(100k+6\right)\left(2^{\frac{k-3}{2}}\right)+\sum_{k=2}^{2n}\left(100k+6\right)\left(2^{\frac{k}{2}}\right)\right)\\ & \;+\left(200n+106\right)3.4^{n}+W_{1}\left(D_{3}\left[n-1\right]\right). \end{array}$$ (3.2.1)

For $x\in V_{2}\left(\rho \right),y\in V_{2}$ , by counting the shortest-path distances between the corresponding vertex pairs, the Wiener contribution becomes $W_{2}\left(D_{3}\left[n\right]\right)=b_{0}.I_{n,1}+\left(\sum_{k=1}^{2n+1}\left(b_{k}\right)I_{n,5k-3}+\sum_{k=2}^{2n}\left(b_{k}\right)I_{n,5k-3}\right)+W_{2}\left(D_{3}\left[n-1\right]\right)$ . By substituting $b_{0}=0$ , $b_{k}=30k-27$ , and using Lemmas 3.1.1, Lemmas 3.1.2, … , Lemmas 3.1.4, we obtain $W_{2}\left(D_{3}\left[n\right]\right)=0.3.2^{n}+\sum_{k=1}^{2n-1}9\left(30k-27\right)\left(2^{n+\frac{k-3}{2}}\right)+\sum_{k=2}^{2n}3\left(30k-27\right)\left(2^{n+\frac{k}{2}}\right)+\left(200n+106\right).3.4^{n}+W_{2}\left(D_{3}\left[n-1\right]\right).$ It can be written as

$$\begin{array}{ll} W_{2}\left(D_{3}\left[n\right]\right) & =3.2^{n}\left(\sum_{k=1}^{2n-1}3\left(30k-27\right)\left(2^{\frac{k-3}{2}}\right)+\sum_{k=2}^{2n}\left(30k-27\right)\left(2^{\frac{k}{2}}\right)\right)\\ & \;+\left(200n+106\right)3.4^{n}+W_{2}\left(D_{3}\left[n-1\right]\right). \end{array}$$ (3.2.2)

Also, if $x\in V_{1}\left(\rho \right),y\in V_{2}$ , then the Wiener index becomes $W_{3}\left(D_{3}\left[n\right]\right)=c_{0}.I_{n,1}+\left(\sum_{k=1}^{2n+1}\left(c_{k}\right)I_{n,5k-3}+\sum_{k=2}^{2n}\left(c_{k}\right)I_{n,5k-3}\right)+W_{3}\left(D_{3}\left[n-1\right]\right)$ . By substituting $c_{0}=9$ , $c_{k}=50k-17$ , and using Lemmas 3.1.1, Lemmas 3.1.2, … , Lemmas 3.1.4, we obtain $W_{3}\left(D_{3}\left[n\right]\right)=9.3.2^{n}+\sum_{k=1}^{2n-1}9\left(50k-17\right)\left(2^{n+\frac{k-3}{2}}\right)+\sum_{k=2}^{2n}3\left(50k-17\right)\left(2^{n+\frac{k}{2}}\right)+\left(200n+106\right).3.4^{n}+W_{3}\left(D_{3}\left[n-1\right]\right).$ It can be written as

$$\begin{array}{ll} W_{3}\left(D_{3}\left[n\right]\right) & =27.2^{n}+3.2^{n}\left(\sum_{k=1}^{2n-1}3\left(50k-17\right)\left(2^{\frac{k-3}{2}}\right)+\sum_{k=2}^{2n}\left(50k-17\right)\left(2^{\frac{k}{2}}\right)\right)\\ & \;+\left(200n+106\right)3.4^{n}+W_{3}\left(D_{3}\left[n-1\right]\right). \end{array}$$ (3.2.3)

Now, by adding Equations 3.2.1, 3.2.2, 3.2.3, the Wiener index of the graph $D_{3}\left[n\right]$ is as follows:

$$W\left(D_{3}\left[n\right]\right)=\left(27\right)3.2^{n}+9.2^{n}\left(\sum_{k=1}^{2n-1}\left(180k-38\right)\left(2^{\frac{k-3}{2}}\right)\right)+3.2^{n}\left(\sum_{k=1}^{2n-1}\left(180k-38\right)\left(2^{\frac{k}{2}}\right)\right)+\left(360n+142\right)3.4^{n}+W_{1}\left(D_{3}\left[n-1\right]\right)+W_{2}\left(D_{3}\left[n-1\right]\right)+W_{3}\left(D_{3}\left[n-1\right]\right)=81.2^{n}+9.2^{n}\left(180\left(\frac{3}{2}+n.2^{n}-2^{n+1}+2^{n-1}\right)-38\frac{2^{n}-1}{2}\right)+3.2^{n}\left(360\left(n-1\right)2^{n+1}+2\right)-38\left(2^{n+1}-2\right)+\left(360n+142\right)3.4^{n}+W\left(D_{3}\left[n-1\right]\right)=81.2^{n}+3.2^{n}\left(\left(360n-360\right)2^{n+1}+720-38.2^{n+1}+76\right)+9.2^{n}\left(180\left(\frac{3}{2}+n2^{n}-2^{n+1}+2^{n-1}\right)-19\left(2^{n}-1\right)\right)+\left(360n+142\right)3.4^{n}+W\left(D_{3}\left[n-1\right]\right)=81\left(2^{n}\right)+3.2^{n}\left(\left(360n-398\right)2^{n+1}+796\right)+9.2^{n}\left(\left(180n-19\right)2^{n}+180\left(2^{n-1}-2^{n+1}\right)+289\right)+3.4^{n}\left(360n+142\right)+W\left(D_{3}\left[n-1\right]\right).$$

Here, $W\left(D_{3}\left[0\right]\right)=507$ . Further computations gives the following equation:

$$W\left(D_{3}\left[n\right]\right)=4860.n4^{n}-4563.4^{n}+5070.2^{n}+W\left(D_{3}\left[n-1\right]\right).$$

This completes the proof.

### 3.3 Computation of the Gutman index of $D_{3}\left[n\right]$

In the next theorem, the Gutman index of the graph $D_{3}\left[n\right]$ is computed.

Theorem 3.3.1 *For positive integers* n≥1 *,*

$$Gut\left(D_{3}\left[n\right]\right)=95169.2^{n-1}+46170.n4^{n}-\frac{86697}{2}.4^{n}+Gut\left(D_{3}\left[n-1\right]\right).$$

Proof. Consider $V\left(D_{3}\left[n\right]\right)=V_{1}\cup V_{2}$ , where $V_{1}$ and $V_{2}$ are the vertices of degrees 2 and 3, respectively, and $V_{1}\left(\rho \right)$ and $V_{2}\left(\rho \right)$ represent the pendent cycle vertices with degree 2 & 3 there correspondingly, as defined earlier. Now, for $x\in V_{1}\left(\rho \right),y\in V_{1}$ , the Gutman index of the graph $D_{3}\left[n\right]$ is as follows:

$$Gut_{1}\left(D_{3}\left[n\right]\right)=2\left(a_{0}.I_{n,1}+\overset{2n+1}{\underset{k=1}{\sum }}\left(a_{k}\right)I_{n,5k-3}+\overset{2n}{\underset{k=2}{\sum }}\left(a_{k}\right)I_{n,5k-3}\right.+Gut_{1}\left(D_{3}\left[n-1\right]\right)).$$

By substituting $a_{0}=18,a_{k}=100k+6$ and using Lemmas 3.1.1, Lemmas 3.1.2, … , Lemmas 3.1.4, we obtain

$$\begin{array}{ll} Gut_{1}\left(D_{3}\left[n\right]\right) & =108.2^{n}+6.2^{n}\left[\overset{2n-1}{\underset{k=1}{\sum }}3\left(100k+6\right)\left(2^{\frac{k-3}{2}}\right)+\overset{2n}{\underset{k=2}{\sum }}\left(100k+6\right)\left(2^{\frac{k}{2}}\right)\right]\\ & \;+\left(200n+106\right)6.4^{n}+2.Gut_{1}\left(D_{3}\left[n-1\right]\right). \end{array}$$ (3.3.1)

For $x\in V_{2}\left(\rho \right),y\in V_{2}$ , the Gutman index using Lemmas 3.1.1, Lemmas 3.1.2, … , Lemmas 3.1.4, becomes

$$Gut_{2}\left(D_{3}\left[n\right]\right)=\frac{9}{2}\left(\overset{2n-1}{\underset{k=1}{\sum }}9\left(30k-27\right)\left(2^{n+\frac{k-3}{2}}\right)+\overset{2n}{\underset{k=2}{\sum }}3\left(30k-27\right)\left(2^{n+\frac{k}{2}}\right)\right.+\left(200n+106\right).3.4^{n}+Gut_{2}\left(D_{3},\left[n-1\right]\right)).$$

It can also be written as

$$\begin{array}{ll} Gut_{2}\left(D_{3}\left[n\right]\right) & =\frac{27}{2}.2^{n}\left[\overset{2n-1}{\underset{k=1}{\sum }}3\left(30k-27\right)\left(2^{\frac{k-3}{2}}\right)+\overset{2n}{\underset{k=2}{\sum }}\left(30k-27\right)\left(2^{\frac{k}{2}}\right)\right]+\left(200n+106\right)\frac{27}{2}.4^{n}\\ & \;+\frac{9}{2}Gut_{2}\left(D_{3}\left[n-1\right]\right). \end{array}$$ (3.3.2)

In addition, if $x\in V_{1}\left(\rho \right),y\in V_{2}$ , then the Gutman index using Lemmas 3.1.1, Lemmas 3.1.2, … , Lemmas 3.1.4, is

$$Gut_{3}\left(D_{3}\left[n\right]\right.=3\left(9.3.2^{n}+\overset{2n-1}{\underset{k=1}{\sum }}9\left(50k-17\right)\left(2^{n+\frac{k-3}{2}}\right)\right.+\overset{2n}{\underset{k=2}{\sum }}3\left(50k-17\right)\left(2^{n+\frac{k}{2}}\right)+\left(200n+106\right).3.4^{n}+Gut_{3}\left(D_{3}\left[n-1\right]\right)).$$

It can be expressed as

$$\begin{array}{ll} Gut_{3}\left(D_{3}\left[n\right]\right) & =81.2^{n}+9.2^{n}\left(\overset{2n-1}{\underset{k=1}{\sum }}3\left(50k-17\right)\left(2^{\frac{k-3}{2}}\right)+\overset{2n}{\underset{k=2}{\sum }}\left(50k-17\right)\left(2^{\frac{k}{2}}\right)\right)\\ & \;+\left(200n+106\right)9.4^{n}+3Gut_{3}\left(D_{3}\left[n-1\right]\right). \end{array}$$ (3.3.3)

Now, by adding Equations 3.3.1, 3.3.2, 3.3.3, the Gutman index of the graph $D_{3}\left[n\right]$ is

$$Gut\left(D_{3}\left[n\right]\right)=189.2^{n}+\frac{19}{2}\left[9.2^{n}\left(180\left(n.2^{n}-2^{n+1}+2^{n-1}+\frac{3}{2}\right)\right)\right.-38\frac{2^{n}-1}{2}+3.2^{n}\left(360\left(n-1\right)2^{n+1}+2\right)-38\left(2^{n+1}-2\right)+\left(360n+142\right)3.4^{n}+Gut\left(D_{3}\left[n-1\right]\right)=189.2^{n}+\frac{19}{2}\left[3.2^{n}\left(\left(360n-360\right)2^{n+1}+720-38.2^{n+1}+76\right)\right]+9.2^{n}\left(180\left(\frac{3}{2}+n2^{n}-2^{n+1}+2^{n-1}\right)-19\left(2^{n}-1\right)\right)+Gut\left(D_{3}\left[n-1\right]\right)]=189.2^{n}+\frac{19}{2}\left[3.2^{n}\left(\left(360n-398\right)2^{n+1}+796\right)\right.+9.2^{n}\left(\left(180n-19\right)2^{n}+180\left(2^{n-1}-2^{n+1}\right)+289\right)+3.4^{n}\left(360n+142\right)+Gut\left(D_{3}\left[n-1\right]\right),$$

where $Gut\left(D_{3}\left[0\right]\right)=\frac{9633}{2}$ . Further computation gives

$$Gut\left(D_{3}\left[n\right]\right)=95169.2^{n-1}+46170.n4^{n}-\frac{86697}{2}.4^{n}+Gut\left(D_{3}\left[n-1\right]\right).$$

This completes the proof.

### 3.4 Computation of the degree distance index of $D_{3}\left[n\right]$

In the next theorem, the degree distance index of the graph $D_{3}\left[n\right]$ is computed.

Theorem 3.4.1 *For positive integers* n≥1 *,*

$$DD\left(D_{3}\left[n\right]\right)=37593.2^{n}+36450.n4^{n}-\frac{68445}{2}.4^{n}+DD\left(D_{3}\left[n-1\right]\right).$$

Proof. Consider $V\left(D_{3}\left[n\right]\right)=V_{1}\cup V_{2}$ , where $V_{1}$ and $V_{2}$ are the vertices of degrees 2 and 3, respectively, and $V_{1}\left(\rho \right)$ and $V_{2}\left(\rho \right)$ represent the pendent cycle vertices with degree 2 & 3 there correspondingly, as defined earlier. Now, for $x\in V_{1}\left(\rho \right),y\in V_{1}$ , the degree distance index of the graph $D_{3}\left[n\right]$ is

$$DD_{1}\left(D_{3}\left[n\right]\right)=2\left(a_{0}.I_{n,1}+\overset{2n+1}{\underset{k=1}{\sum }}\left(a_{k}\right)I_{n,5k-3}+\overset{2n}{\underset{k=2}{\sum }}\left(a_{k}\right)I_{n,5k-3}+DD_{1}\left(D_{3}\left[n-1\right]\right)\right).$$

By substituting the values $a_{0}=18,a_{k}=100k+6$ and using Lemmas 3.1.1, Lemmas 3.1.2, … ,Lemmas 3.1.4, we obtain

$$\begin{array}{ll} DD_{1}\left(D_{3}\left[n\right]\right) & =108.2^{n}+6.2^{n}\left(\overset{2n-1}{\underset{k=1}{\sum }}3\left(100k+6\right)\left(2^{\frac{k-3}{2}}\right)+\overset{2n}{\underset{k=2}{\sum }}\left(100k+6\right)\left(2^{\frac{k}{2}}\right)\right)\\ & \;+\left(200n+106\right)6.4^{n}+2.DD_{1}\left(D_{3}\left[n-1\right]\right). \end{array}$$ (3.4.1)

For $x\in V_{2}\left(\rho \right),y\in V_{2}$ , by counting the shortest-path distances between the corresponding vertex pairs, the degree distance contribution using Lemmas 3.1.1, Lemmas 3.1.2, … , Lemmas 3.1.4, becomes

$$DD_{2}\left(D_{3}\left[n\right]\right)=3\left(\overset{2n-1}{\underset{k=1}{\sum }}9\left(30k-27\right)\left(2^{n+\frac{k-3}{2}}\right)+\overset{2n}{\underset{k=2}{\sum }}3\left(30k-27\right)\left(2^{n+\frac{k}{2}}\right)\right)+\left(200n+106\right).3.4^{n}+DD_{2}\left(D_{3}\left[n-1\right]\right)).$$

It can be written as

$$\begin{array}{ll} DD_{2}\left(D_{3}\left[n\right]\right) & =9.2^{n}\left(\overset{2n-1}{\underset{k=1}{\sum }}3\left(30k-27\right)\left(2^{\frac{k-3}{2}}\right)+\overset{2n}{\underset{k=2}{\sum }}\left(30k-27\right)\left(2^{\frac{k}{2}}\right)\right)\\ & \;+\left(200n+106\right)9.4^{n}+3.DD_{2}\left(D_{3}\left[n-1\right]\right). \end{array}$$ (3.4.2)

In addition, if $x\in V_{1}\left(\rho \right),y\in V_{2}$ , then the degree distance index using Lemmas 3.1.1, Lemmas 3.1.2, …and , Lemmas 3.1.4, is:

$$DD_{3}\left(D_{3}\left[n\right]\right)=\frac{5}{2}\left(9.3.2^{n}+\overset{2n-1}{\underset{k=1}{\sum }}9\left(50k-17\right)\left(2^{n+\frac{k-3}{2}}\right)\right.+\overset{2n}{\underset{k=2}{\sum }}3\left(50k-17\right)\left(2^{n+\frac{k}{2}}\right)+\left(200n+106\right).3.4^{n}+DD_{3}\left(D_{3}\left[n-1\right]\right)).$$

It can be expressed as

$$\begin{array}{ll} DD_{3}\left(D_{3}\left[n\right]\right) & =\frac{135}{2}.2^{n}+\frac{15}{2}.2^{n}\left(\overset{2n-1}{\underset{k=1}{\sum }}3\left(50k-17\right)\left(2^{\frac{k-3}{2}}\right)+\overset{2n}{\underset{k=2}{\sum }}\left(50k-17\right)\left(2^{\frac{k}{2}}\right)\right)+\frac{5}{2}.4^{n}\left(200n+106\right)\\ & \;+\frac{5}{2}DD_{3}\left(D_{3}\left[n-1\right]\right). \end{array}$$ (3.4.3)

Now, by adding Equations 3.4.1, 3.4.2, 3.4.3, the degree distance index of the graph $D_{3}\left[n\right]$ is

$$DD\left(D_{3}\left[n\right]\right)=\frac{351}{2}.2^{n}+\frac{15}{2}\left.(3.2^{n}\left(\left(360n-360\right)2^{n+1}+720-38.2^{n+1}+76\right)\right.+9.2^{n}\left(180\left(\frac{3}{2}+n2^{n}-2^{n+1}+2^{n-1}\right)-19\left(2^{n}-1\right))\right)+DD_{1}\left(D_{3}\left[n-1\right]\right)+DD_{2}\left(D_{3}\left[n-1\right]\right)+DD_{3}\left(D_{3}\left[n-1\right]\right)=\frac{351}{2}.2^{n}+\frac{15}{2}\left[3.2^{n}\left(\left(360n-398\right)2^{n+1}+796\right)\right]+9.2^{n}\left(\left(180n-19\right)2^{n}+180\left(2^{n-1}-2^{n+1}\right)+289\right)+3.4^{n}\left(360n+142\right)+DD\left(D_{3}\left[n-1\right]\right),$$

where $DD\left(D_{3}\left[0\right]\right)=\frac{7605}{2}$ . By further computing, it becomes

$$DD\left(D_{3}\left[n\right]\right)=37593.2^{n}+36450.n4^{n}-\frac{68445}{2}.4^{n}+DD\left(D_{3}\left[n-1\right]\right)$$

This completes the proof.

### 3.5 Computation of the Wiener polarity index

From the construction of the graph $D_{3}\left[n\right]$ , it is easy to observe that $W_{P}\left(D_{3}\left[2\right]\right)=21\left(3\right)+24\left(8\right)=63+192=255$ . More generally, we obtain the following result.

Theorem 3.5.1 *For* n≥1 *,*

$$W_{P}\left(D_{3}\left[n\right]\right)=33.2^{n+1}-9.$$

Proof. For n≥1 , each newly attached hexagon contributes three pairs of vertices at distance 3 inside the hexagon and eight pairs at distance 3 between adjacent hexagons at the same stage. Between consecutive stages, exactly 21 new pairs appear at distance 3, and there is no pair of vertices at distance 3 between the hexagons of stages j−2 and j . Summing these contributions recursively yields $W_{P}\left(D_{3}\left[n\right]\right)=W_{P}\left(D_{3}\left[2\right]\right)+3.\left(24+48+96+\cdots +2^{n-2}\right)+8.\left(24+48+96+\cdots +2^{n-2}\right)=9.\left(2^{n+1}-1\right)+96.\left(2^{n-1}\right)=9.\left(2^{n+1}-1\right)+24.\left(2^{n+1}\right)=33.\left(2^{n+1}\right)-9$ .

### 3.6 Verification for small values of n

Table 2 compares the closed formulae with the recursive relations for n=0,1,2,3,…,8 . The agreement confirms the analytical expressions for the initial stages.

**TABLE 2:** Verification of index formulae of the graph $D_{3}\left[n\right]$ for small values of n

n	$DD\left(D_{3}\left[n\right]\right)$	$Gut\left(D_{3}\left[n\right]\right)$	$W\left(D_{3}\left[n\right]\right)$	$W_{P}\left(D_{3}\left[n\right]\right)$
0	$\frac{7605}{2}$	$\frac{9633}{2}$	507	57
1	$\frac{175797}{2}$	$\frac{222543}{2}$	11835	123
2	$\frac{1714221}{2}$	$\frac{2170947}{2}$	114627	255
3	$\frac{11932029}{2}$	$\frac{15112971}{2}$	796275	519
4	$\frac{70262685}{2}$	$\frac{88997403}{2}$	4685907	1047
5	$\frac{375828957}{2}$	$\frac{476045883}{2}$	25058835	2103
6	$\frac{1891880541}{2}$	$\frac{2396373627}{2}$	126132627	4215
7	$\frac{9140856669}{2}$	$\frac{11578401531}{2}$	609405075	8439
8	$\frac{42895087965}{2}$	$\frac{54333744123}{2}$	2859701907	16887

## 4 Computation of degree-based graph entropy

Let G be a graph with the vertex set V(G) and m=|E(G)|>0 . Let D(G) denote the set of vertex degrees occurring in G , and for each r∈D(G) , let $V_{r}=\left\{v\in V\left(G\right):\deg\left(v\right)=r\right\}$ . The degree-distribution entropy of G , denoted by Ent(G) , is defined by

$$Ent\left(G\right)=-\underset{r\in D\left(G\right)}{\sum }p_{r}\log_{2}p_{r},\;p_{r}=\frac{\left|V_{r}\right|}{\left|V\left(G\right)\right|}.$$ (4.0.1)

Here, $p_{r}$ is the probability that a randomly chosen vertex has degree r . This is the Shannon entropy of the degree sequence (Shannon, 1948). Since $0<p_{r}<1$ for each contributing term, it follows that Ent(G)≥0 . For $D_{3}\left[n\right]$ , only degrees 2 and 3 occur. Using the vertex counts from Section 3.1, we obtain

$$p_{2}=\frac{\left|V_{1}\right|}{\left|V\left(D_{3}\left[n\right]\right)\right|}=\frac{27\cdot 2^{n}-12}{39\cdot 2^{n}-20},\;p_{3}=\frac{\left|V_{2}\right|}{\left|V\left(D_{3}\left[n\right]\right)\right|}=\frac{12\cdot 2^{n}-8}{39\cdot 2^{n}-20}.$$

Therefore the Equation 4.0.1 can be written as below:

$$Ent\left(D_{3}\left[n\right]\right)=-p_{2}\log_{2}p_{2}-p_{3}\log_{2}p_{3}.$$ (4.0.2)

Theorem 4.0.1 *For every* n≥0 *, the degree-distribution entropy of* $D_{3}\left[n\right]$ *is given in* Equation 4.0.2 *. In particular,* $Ent\left(D_{3}\left[n\right]\right)>0$ *for all* n≥0 .

Proof. As n increases, the degree proportions approach $p_{2}\to \frac{9}{13}$ and $p_{3}\to \frac{4}{13}$ , so the entropy converges monotonically to

$$Ent\left(D_{3}\left[n\right]\right)\to -\frac{9}{13}\log_{2}\;\left(\frac{9}{13}\right)-\frac{4}{13}\log_{2}\;\left(\frac{4}{13}\right)\approx 0.8905.$$

This convergence reflects the stabilization of the degree distribution in the recursive construction.

**FIGURE 3:**
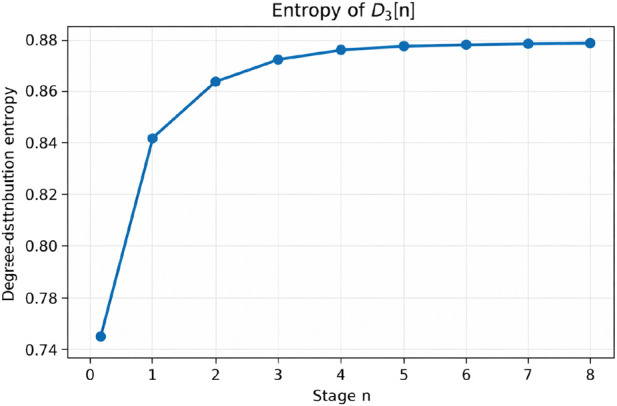
Graphical representation of entropy values.

Figure 3 displays the entropy values. As n increases, the curve increases monotonically and approaches the limiting value given above.

## 5 Graphical comparison of the indices of the graph

**FIGURE 4:**
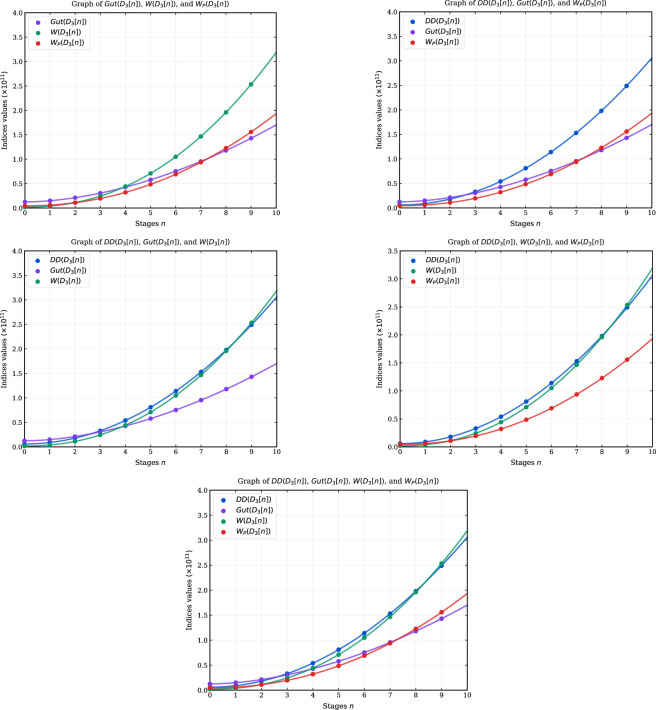
Graphical comparison of $Gut\left(D_{3}\left[n\right]\right)$ , $W\left(D_{3}\left[n\right]\right)$ , $DD\left(D_{3}\left[n\right]\right)$ , and $W_{P}\left(D_{3}\left[n\right]\right)$ . Separate panels are shown because the indices have different magnitudes; the combined panel should be interpreted on a logarithmic scale.

In this section, we analyze the behavior of $Gut\left(D_{3}\left[n\right]\right)$ , $W\left(D_{3}\left[n\right]\right)$ , $DD\left(D_{3}\left[n\right]\right)$ , and $W_{P}\left(D_{3}\left[n\right]\right)$ . As these indices increase rapidly and have very different magnitudes, Table 2 provides exact values. Figure 4 presents separate panels for each index; the combined panel should be read on a logarithmic scale.

The Wiener index is known to correlate with the boiling points of alkane molecules (Wiener, 1947). For nanostar dendrimers, the computed indices are interpreted here as theoretical structural descriptors that describe size, branching, and distance distribution.

### 5.1 Structural interpretation

The computed indices describe the recursive growth of $D_{3}\left[n\right]$ from a graph-theoretic viewpoint. The Wiener index measures the total spread of distances, the Gutman and degree distance indices combine distances with vertex degrees, and the Wiener polarity index counts vertex pairs at distance 3. The entropy quantifies the variability of the degree sequence. These quantities are theoretical structural descriptors; they may be useful in future QSPR or QSAR studies once suitable chemical data become available.

## 6 Conclusion

In this study, we primarily aim to contribute by providing a detailed structural feature analysis and evaluation of Nanostar dendrimers using graph theory concepts. Our computation of indices such as degree distance, Gutman, Wiener, and Wiener polarity indices offers valuable insights into the structural complexities of these dendrimers and can be applied to materials science and nanomedicine, among other areas, to optimize their functionalities. In addition, details concerning the structural characteristics of nanostar dendrimers can be obtained from the graphical analysis of these index values. Improving our understanding of the complexity and diversity of the graph can also be achieved by computing the graph’s entropy. Calculation of these indices helps us understand and characterize the structural and chemical characteristic of these molecules. Each of these indices offers distinct perspectives into different aspects of the graph or molecular arrangements.

Determining the degree distance for nanostar dendrimers provide insights into the vertex degree distribution, crucial for understanding the branching patterns and topological connectivity within the molecule. Determining the Gutman index for the nanostar dendrimer graph can yield insights into the electronic structure and overall topology of the molecule. When evaluating structural alterations in a group of related molecules or comparing various dendrimers, the Wiener index can be used to quantify the branching complexity and the size of the molecule. Understanding the overall polarity of the molecule is important for comprehending its potential uses across variety of fields. This can be achieved by determining the Wiener polarity index of the nanostar dendrimer graph.

We emphasize that these results are theoretical structural descriptors. Although such indices are widely used in mathematical chemistry, no direct QSAR or QSPR validation is performed in the present article. Accordingly, the formulae should be viewed as a basis for future experimental or computational correlation studies involving nanostar dendrimers. The information content and structural disorder of nanostar dendrimers can be measured by determining their entropy. This understanding can significantly enhance insights into the molecular behavior, stability, and potential applications of these dendrimers across various fields, such as materials science, drug delivery, and nanotechnology.

In conclusion, the recursive formulae for $W\left(D_{3}\left[n\right]\right)$ , $Gut\left(D_{3}\left[n\right]\right)$ , $DD\left(D_{3}\left[n\right]\right)$ , $W_{P}\left(D_{3}\left[n\right]\right)$ , and $Ent\left(D_{3}\left[n\right]\right)$ , together with the verification for small n and the graphical comparisons, provide a reproducible framework for studying the topological complexity of $D_{3}\left[n\right]$ . These indices are theoretical structural descriptors and may support future comparative or QSPR studies when suitable chemical data become available.

## Data Availability

The original contributions presented in the study are included in the article/supplementary material; further inquiries can be directed to the corresponding authors.
